# Tests on the Accuracy and Scalability of the Full-Potential DFT Method Based on Multiple Scattering Theory

**DOI:** 10.3389/fchem.2020.590047

**Published:** 2020-12-04

**Authors:** Peiyu Cao, Jun Fang, Xingyu Gao, Fuyang Tian, Haifeng Song

**Affiliations:** ^1^State Key Laboratory for Advanced Metals and Materials, University of Science and Technology Beijing, Beijing, China; ^2^State Key Laboratory of Nonlinear Mechanics, Institute of Mechanics, Chinese Academy of Sciences, Beijing, China; ^3^Laboratory of Computational Physics, Institute of Applied Physics and Computational Mathematics, Beijing, China; ^4^Institute for Applied Physics, University of Science and Technology Beijing, Beijing, China

**Keywords:** first principles, Korringa–Kohn–Rostoker (KKR), multiple scattering theory (MST), full potential, elastic constants

## Abstract

We investigate a reduced scaling full-potential DFT method based on the multiple scattering theory (MST) code MuST, which is released online (https://github.com/mstsuite/MuST) very recently. First, we test the accuracy by calculating structural properties of typical body-centered cubic (BCC) metals (V, Nb, and Mo). It is shown that the calculated lattice parameters, bulk moduli, and elastic constants agree with those obtained from the VASP, WIEN2k, EMTO, and Elk codes. Second, we test the locally self-consistent multiple scattering (LSMS) mode, which achieves reduced scaling by neglecting the multiple scattering processes beyond a cut-off radius. In the case of Nb, the accuracy of 0.5 mRy/atom can be achieved with a cut-off radius of 20 Bohr, even when small deformations are imposed on the lattice. Despite that the calculation of valence states based on MST exhibits linear scaling, the whole computational procedure has an overall scaling of about $O\left(N^{1.6}\right)$, due to the fact that the updating of Coulomb potential scales almost as $O\left(N^{2}\right)$. Nevertheless, it can be still expected that MuST would provide a reliable and accessible way to large-scale first-principles simulations of metals and alloys.

## 1. Introduction

Kohn–Sham density functional theory (KS-DFT) (Kohn and Sham, 1965) transforms the many-body problem to a non-interacting system and has been widely used in modern first-principles calculations. Although many computational schemes are developed to solve the Kohn–Sham equation (Kohn and Sham, 1965) for the ground-state properties, the Korringa–Kohn–Rostoker Green's function (KKR-GF) method (Korringa, 1947; Kohn and Rostoker, 1954), also known as multiple scattering theory (MST), provides equivalent information by the single-particle GF (Economou, 2006). In the MST approach, the system is divided into non-overlapping atomic regions as a set of scatterers. To solve the single-site scattering problem, one numerically determines the angular momentum and energy-dependent solutions of the radial Schrödinger equation for a given potential. The coherent matching of the single-site solutions can be achieved if and only if the incoming wave of an atomic site is identical to the superposition of the outgoing waves from all other scatterers. This viewpoint not only gives access to the Kohn–Sham eigenstates but also to the single-electron GF of the system, which leads to the modern KKR-GF method.

The survey (Aarons et al., 2016) suggests that KKR-GF or MST method remains important for large-scale metallic systems. The favorable scaling in MST is attributed to the fact that the electron density, which is the fundamental quantity in DFT, can be obtained from the site-diagonal blocks of the scattering path matrix. And the site-diagonal block of the scattering path matrix for a particular atom can be solved with sufficient accuracy by considering only the electronic multiple scattering processes in a finite-sized region centered at this atom. This region is referred to as the local interaction zone (LIZ), which is originally introduced in the locally self-consistent multiple-scattering method (LSMS) (Wang et al., 1995). Base on the central idea of the LSMS method, the locally self-consistent GF (LSGF) approach (Abrikosov et al., 1996, 1997) can choose judiciously the effective medium to decrease the LIZ size. In particular, the linear scaling has been achieved in LSMS with muffin-tin approximation and in LSGF with tight-binding linear muffin-tin orbital (TB-LMTO) basis. It should be mentioned that besides the MST-based methods, other approaches to reduced scaling DFT methods for metallic systems have also been developed in recent years Pratapa et al. (2016), Suryanarayana et al. (2018), Aarons and Skylaris (2018), Mohr et al. (2018).

There is a trend toward the full-potential (FP) MST in which no shape approximation is assumed for the potential. Many questions in materials science, for example, on complex defects, interfaces, dislocations, as well as nanostructures, come to a great demand for the reduced scaling FP method. KKRnano, a massively parallel DFT package based on MST, has been developed and optimized for thousands of atoms without a compromise on the FP accuracy (Thiess et al., 2012). And this package has been applied to study the role of the vacancy clusters in metal-insulator transitions (Zhang et al., 2012).

However, most MST simulation packages are in-house, which impedes the application of MST as a powerful tool for large scale or disordered systems. Recently, the MuST package, an *ab initio* calculation software package based on FP MST (Rusanu et al., 2011), is open to public and is free to download online (https://github.com/mstsuite) under a BSD 3-clause license. We focus on the MST part in the MuST package, which not only provides features for calculating physical properties of materials but also serves as a platform for implementing and testing the numerical algorithms. At present, the MuST package is capable of performing the following calculations: (1) muffin-tin approximation, (2) FP method, (3) coherent potential approximation (CPA), and (4) LSMS method. And the fully relativistic MST by solving the Dirac equation has been implemented in MuST Liu et al. (2016, 2018). For such a newly released package, it is prerequisite to perform systematic tests both on the accuracy and efficiency.

A reliable FP method can be used to exactly capture the small energy difference for the lattice distortion or deformation. According to the elastic theory, we deform the crystalline cell to the distorted lattice structures and then calculate their energies. The small energy change with the lattice deformation can be used to calculate the elastic constants (Vitos, 2007). Asato et al. (1999) investigated total energy calculations for metals and semiconductors based on the FP MST method. But few work pay attention to validate the elastic properties based on MST, which is fundamental for applying MST to study the structural properties of materials. Considering the anomalies behavior of deformations in body-centered cubic (BCC) V and Nb (Nagasako et al., 2010; Dezerald et al., 2016), we employ the different *ab initio* methods including the FP MST method in MuST package to calculate the elastic constants of V, Nb, and Mo. By comparing with results of available experiments and other popular first-principles packages, we investigate the accuracy of the FP MST method in MuST package.

To estimate the parallel scalability, we carried out strong and weak scaling tests of the FP LSMS method in MuST package. It is seen that the LSMS method exhibits a good strong scalability. This is due to the two-level parallelism over atoms and energy points implemented in MuST package. However, in the weak scaling test, the overall computational procedure is not linear scaling, which seems to be inconsistent with the O(N) scaling of the muffin-tin LSMS proved in previous work (Wang et al., 1995). By analyzing the implementation scheme, we attribute it to the difference in updating the Coulomb potential between the muffin-tin approximation and the FP method. While the solution of eigenvalue problems is avoided in the MST method, the calculation of Coulomb potential could become the performance bottleneck in large-scale first-principles simulations. For example, PRinceton Orbital-Free Electronic Structure Software (PROFESS) (Ho et al., 2008; Hung et al., 2010; Chen et al., 2015) suggested that about 70% of computation time was spent on fast Fourier transforms (FFTs) to calculate the kinetic and electron–electron Coulomb interaction terms. PROFESS features plane-wave basis set and has been optimized for peta-scale computing (Chen et al., 2016). The calculation of MST is based on angular momentum expansion and new algorithms should be developed to optimize the overall scaling of the FP MST method.

The rest of this paper is arranged as follows. In section 2, we introduce the MST method and its LSMS variant. In section 3, we investigate the accuracy by calculating the equation of state (EOS) and elastic constants of typical BCC metals. In section 4, we test and analyze the scalability of the FP LSMS method. In section 5, some concluding remarks are drawn.

## 2. Methodology

### 2.1. MST Method

The term MST method in this manuscript refers to the modern version of the KKR method, that is, the KKR-GF method. The central quantity to be computed in the MST method changes from the Kohn–Sham orbitals in band theory methods to the one-electron GF, which can be defined as solutions of the following differential equation (Economou, 2006) (non-spin polarized cases assumed and atomic units ℏ = 1 and *m*_*e*_ = 1/2 used):

(1) $$\begin{array}{r} \left\{z+\nabla^{2}-V_{\text{eff}}\left[\rho \right]\left(r\right)\right\}G\left(r,r^{\prime };z\right)=\delta \left(r-r^{\prime }\right), \end{array}$$

where *V*_eff_ is the Kohn–Sham effective potential under exchange-correlation approximations like the local density approximation (LDA) or the generalized gradient approximation (GGA), ρ(***r***) is the electron density, and *z* ≡ ϵ + *ıη* is a complex variable. If *z* is real and ϵ belongs to the continuous spectrum of $-\nabla^{2}+V_{\text{eff}}\left[\rho \right]$, *G*(***r***, ***r***′; ϵ) is not well-defined and one may define the retarded GF

(2) $$\begin{array}{r} G^{+}\left(r,r^{\prime };\epsilon \right)\equiv \underset{\eta \to 0^{+}}{\lim}G\left(r,r^{\prime };\epsilon +ı\eta \right). \end{array}$$

In the following, the superscript + will be omitted. Once the GF is known, the valence electron density can be obtained by (Gonis and Butler, 2000; Economou, 2006; Faulkner et al., 2018)

(3) $$\begin{array}{r} \rho \left(r\right)=-\frac{2}{\pi }\text{Im}\int_{\epsilon_{B}}^{\epsilon_{F}}G\left(r,r;\epsilon \right)\text{d}\epsilon , \end{array}$$

where ϵ_*F*_ is the Fermi energy, the bottom energy ϵ_*B*_ is chosen between the highest core-state energy and the valence band, and the factor 2 accounts for the number of electron spins. The energy integration in Equation (3) can be carried out along a contour in the complex energy plane so that only few tens of energy points are needed. Other physical quantities like the density of states (DOS) can also be obtained from the GF (Gonis and Butler, 2000; Economou, 2006; Faulkner et al., 2018).

The MST method provides a convenient access to the GF. In the MST method, atoms in the system are considered as scattering centers of which the scattering properties are described by the so-called single-site scattering *t*-matrix (Gonis and Butler, 2000; Faulkner et al., 2018). The space is divided into non-overlapping cells Ω_*n*_ centered at atomic positions ***R***_*n*_, where *n* is the index of atoms in the system. In the vicinity of atomic site *n*, it is proved that the GF can be expressed as (Faulkner and Stocks, 1980; Gonis and Butler, 2000; Sébilleau, 2000; Zabloudil et al., 2006)

(4) $$\begin{array}{r} G\left(r_{n},r_{n};\epsilon \right)=\underset{LL^{\prime }}{\sum }Z_{L}^{n}\left(r_{n};\epsilon \right)\tau_{LL^{\prime }}^{nn}\left(\epsilon \right)Z_{L^{\prime }}^{n\times }\left(r_{n};\epsilon \right)\\ \;-\underset{L}{\sum }Z_{L}^{n}\left(r_{n};\epsilon \right)J_{L}^{n\times }\left(r_{n};\epsilon \right), \end{array}$$

where *L* is the combined index of angular momentum quantum number *l* and magnetic quantum number *m*, ***r***_*n*_ ≡ ***r*** − ***R***_*n*_ the relative coordinate, $Z_{L}^{n}\left(r_{n};\epsilon \right)$ and $J_{L}^{n}\left(r_{n};\epsilon \right)$ regular and irregular solutions of the single-site problem in cell *n* for momentum *L* and energy ϵ, and $\tau_{LL^{\prime }}^{nn}\left(\epsilon \right)$ site-diagonal matrix elements of the scattering path operator τ^*nm*^(ϵ) in the angular momentum representation. The × symbol in Equation (4) means that we take the complex conjugate of the spherical harmonics and keep remaining parts of the function unchanged.

The scattering path operator τ^*nm*^(ϵ) describes all possible scattering events of electron states with energy ϵ between atomic sites *n* and *m*. In the angular momentum representation, the corresponding scattering path matrix is given by (Gonis and Butler, 2000; Zabloudil et al., 2006)

(5) $$\begin{array}{l} {\underline{\tau }}^{nm}={\underline{t}}^{n}\delta_{nm}+{\underline{t}}^{n}{\underline{G}}_{0}^{nm}(1-\delta_{nm}){\underline{t}}^{m}+{\underline{t}}^{n}\sum_{k\neq n}{\underline{G}}_{0}^{nk}{\underline{t}}^{k}{\underline{G}}_{0}^{km}(1-\delta_{km}){\underline{t}}^{m}\\ \;+{\underline{t}}^{n}\sum_{k\neq n}{\underline{G}}_{0}^{nk}{\underline{t}}^{k}\sum_{j\neq k}{\underline{G}}_{0}^{kj}{\underline{t}}^{j}{\underline{G}}_{0}^{jm}(1-\delta_{jm}){\underline{t}}^{m}+\ldots \\ \;={\underline{t}}^{n}\delta_{nm}+{\underline{t}}^{n}\sum_{k\neq n}{\underline{G}}_{0}^{nk}{\underline{\tau }}^{km}, \end{array}$$

where the underline symbol indicates matrices with respect to the angular momentum index *L*, *t*^*n*^(ϵ) is the single site scattering *t*-matrix associated with site *n*, and ${\underline{G}}_{0}^{nm}(\epsilon )$ is the free-electron propagator matrix in the angular momentum representation, also known as KKR structure constant matrix, that describes the propagation of a free electron with energy ϵ from site *n* to site *m*. Note that we have omitted the dependence on energy ϵ in Equation (5) for a compact expression.

In the case of a finite system with *N* atoms, it is seen from the second equation in Equation (5) that the scattering path matrix can be computed directly by a matrix inversion:

(6) $${\underline{\tau }}^{nm}(\epsilon )={\left(\begin{array}{ccccc} {\left[{\underline{t}}^{1}(\epsilon )\right]}^{-1} & -{\underline{G}}_{0}^{12}(\epsilon ) & -{\underline{G}}_{0}^{13}(\epsilon ) & \cdots & -{\underline{G}}_{0}^{1N}(\epsilon )\\ -{\underline{G}}_{0}^{21}(\epsilon ) & {\left[{\underline{t}}^{2}(\epsilon )\right]}^{-1} & -{\underline{G}}_{0}^{23}(\epsilon ) & \cdots & -{\underline{G}}_{0}^{2N}(\epsilon )\\ -{\underline{G}}_{0}^{31}(\epsilon ) & -{\underline{G}}_{0}^{32}(\epsilon ) & {\left[{\underline{t}}^{3}(\epsilon )\right]}^{-1} & \cdots & -{\underline{G}}_{0}^{3N}(\epsilon )\\ \vdots & \vdots & \vdots & \ddots & \vdots \\ -{\underline{G}}_{0}^{N1}(\epsilon ) & -{\underline{G}}_{0}^{N2}(\epsilon ) & -{\underline{G}}_{0}^{N3}(\epsilon ) & \cdots & {\left[{\underline{t}}^{N}(\epsilon )\right]}^{-1} \end{array}\right)}_{nm}^{-1},$$

where the subscript *nm* on the right hand side indicates the block at the *n*th row and *m*th column of the big matrix after the inversion has been taken. In the case of periodic systems, the equation in Equation (5) for the scattering path matrix can be solved by the lattice Fourier transform, leading to (we assume that there is only one atom in the unit cell):

(7) $${\underline{\tau }}^{nm}(\epsilon )=\frac{1}{\Omega_{\text{BZ}}}\int_{\Omega_{\text{BZ}}}[\underline{t}{\left(\epsilon \right)}^{-1}-{\underline{G}}_{0}(k,\epsilon ){]}^{-1}{\text{e}}^{ık\cdot (R_{n}-R_{m})}\text{d}k,$$

where Ω_BZ_ is the first Brillouin zone and *G*_0_(***k***, ϵ) is the lattice Fourier transform of ${\underline{\underline{G}}}_{0}(\epsilon )$ (the double underline indicates matrices with respect to the angular momentum index and the atomic site index) (Gonis and Butler, 2000; Zabloudil et al., 2006).

### 2.2. LSMS Method

As described above, the MST method makes unnecessary the calculation of the Kohn–Sham orbitals, and consequently the time-consuming procedure for diagonalization and orthogonalization in the conventional KS-DFT calculations can be entirely avoided. The only global operation required by computing the GF is to obtain the scattering path matrix by an inversion of the matrix in Equation (6). Since the size of the matrix is proportional to the number of atoms in the unit cell, the MST method still suffers from cubic scaling limitation.

To reduce the scaling of the procedure, we can calculate the *n*th site-diagonal block of the scattering path matrix τ^*nn*^ by neglecting the multiple scattering processes that involve atoms beyond some cut-off radius *R*_LIZ_ from atomic site *n*. This is based on the observation that the scattering processes involving far away atoms have little influence on the electronic scattering behavior in the vicinity of atomic site *n*, which is the essence of the LSMS method. The region within distance *R*_LIZ_ from the central atom is called the LIZ. If there are *M* atoms in the LIZ, the solution of the multiple scattering problem scales as $O\left(NM^{3}\right)$, where *N* is the total number of atoms. Consequently, the LSMS method is expected to exhibit the linear scaling in *N* with a prefactor determined by *M* and the number of basis functions.

### 2.3. Coherent Potential Approximation

Due to the convenient access to the GF, the MST method plays a prominent role in first-principles alloy theory, in which a novel candidate is the CPA (Soven, 1967; Taylor, 1967; Gyorffy, 1972; Ruban and Abrikosov, 2008). The CPA is designed to obtain an ordered effective medium to describe properties of the multi-component random alloy. The scattering path operator of the CPA effective medium, denoted by τ_CPA_, is determined by the following self-consistency condition (two-component alloy as the example):

(8) $$\begin{array}{r} \tau_{\text{CPA}}=C_{A}\tau_{A}+C_{B}\tau_{B}, \end{array}$$

where τ_*A*(*B*)_ is the scattering path operator of the auxiliary system constructed by replacing the central site in the ordered effective medium system by the alloy component A(B). Within the single-site approximation, it can be proved that the GF of the CPA effective medium system is equal to the targeted ensemble averaged GF (Faulkner, 1982; Ebert et al., 2011). The CPA condition in Equation (8) needs to be reformulated into a proper expression to be suited for numerical applications (Faulkner, 1982; Turek et al., 1997).

## 3. Test on Accuracy

In this section, we investigate the accuracy of the FP MST method implemented in the MuST package by comparing equilibrium bulk properties and elastic constants with those calculated by the WIEN2k, EMTO, and VASP codes.

### 3.1. Calculation Details

In order for a meaningful comparison, we used the Perdew–Burke–Ernzerhof (PBE) exchange-correlation functional (Perdew et al., 1996) in all our calculations, and carried out convergence tests to determine the numerical parameters for each code. The relativistic effect of the core electrons was treated via the default scheme in each package. In the following, we enumerate the detailed settings of numerical parameters.

#### 3.1.1. MuST

The uniform grid for the computation of the Coulomb potential was chosen as 64 × 64 × 64. The Monkhorst–Pack ***k***-point mesh was set to be 21 × 21 × 21 in all the KKR tests. The break condition for the electronic SCF (self-consistent field) iterations was that differences in the total energy and the potential are smaller than 5 × 10^−8^ Ry and 10^−7^ Ry, respectively. The maximum angular momentum used in the expansion of the wave functions and the GFs was set to *l*_max_ = 4. The number of radial grid points from the atomic center to the muffin-tin radius was chosen to be 2001, which is sufficiently accurate for solving the single-site scattering problem.

#### 3.1.2. WIEN2k

The WIEN2k package (Blaha et al., 2020) implements an FP linearized augmented plane-wave (LAPW) method. No shape approximations have been made on the potential and charge density inside the muffin-tin spheres and in the interstitial region. In our calculations, the muffin-tin sphere radius was fixed as 2.50 Bohr, the cutoff parameter *R*_MT_·*K*_max_ was chosen to be 8.00, and the plane-wave expansion cutoff *G*_max_ was set as 14.00 Ry. And a 15 × 15 × 15 Monkhorst–Pack ***k***-point mesh was used for the Brillouin zone sampling. The chosen *R*_MT_ · *K*_max_ and ***k***-mesh ensure that errors in the total energies of the deformed structures are converged to 10^−4^ Ry in elastic constant calculations.

#### 3.1.3. EMTO

The EMTO package implements the so-called exact muffin-tin orbitals method, in which different from former muffin-tin methods, the single-electron states are calculated exactly for the optimized overlapping muffin-tin (OOMT) potential. We refer the readers to Vitos et al. (2001), Vitos (2001), and Vitos (2007) for the detailed theory and applications of the EMTO method. In our calculations, the EMTO basis set including *s*, *p*, *d*, and *f* orbitals was used in combination with soft-core approximation. For the integration over energy in the complex plane, we used 24 points along a semicircular contour. The Brillouin zone was sampled by a 21 × 21 × 21 Monkhorst–Pack ***k***-point mesh to make the total energies of the deformed structures converge up to 3 × 10^−5^ Ry.

#### 3.1.4. VASP

The Vienna *ab initio* simulation package (VASP) (Kresse and Furthmüller, 1996a,b; Kresse and Joubert, 1999) describes the electron-ion interactions by the projector-augmented wave (PAW) method. In our calculations, the kinetic energy cutoff for the plane-wave basis set was 400 eV. A 15 × 15 × 15 Monkhorst–Pack ***k***-point mesh was used for the Brillouin zone sampling. And the SCF convergence criterion was set to be 10^−7^ Ry.

### 3.2. Equation of State

The lattice parameter *a*, bulk modulus *B*, and pressure derivative of the bulk modulus *B*′ have been commonly used for accuracy assessments of DFT codes and (pseudo)potential libraries (Kucukbenli et al., 2014; Lejaeghere et al., 2014, 2016). These structural properties can be extracted from the EOS for a solid. For instance, in a Morse type of EOS, the total energy is fitted by an exponential function with four parameters (*D*_0_, γ, *a*_0_, and *E*_0_)

(9) $$\begin{array}{r} E\left(a\right)=D_{0}e^{-\gamma \left(\frac{a}{a_{0}}-1\right)}-2D_{0}e^{-\frac{\gamma }{2}\left(\frac{a}{a_{0}}-1\right)}+E_{0}. \end{array}$$

Then *a*_0_, *B*_0_, and *B*′ can be derived from the Morse function and used to assess the accuracy of DFT codes under investigation.

**Figure 1:**
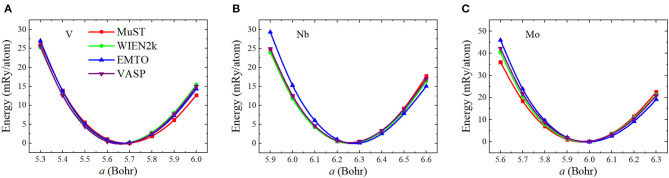
(Color online) Equation of states (the total energy per atom vs. lattice parameter) for body-centered cubic (BCC) V **(A)**, Nb **(B)**, and Mo **(C)**. The total energies have been shifted so that all *E*(*a*) points with the lowest energy are adjusted to zero.

**Figure 2:**
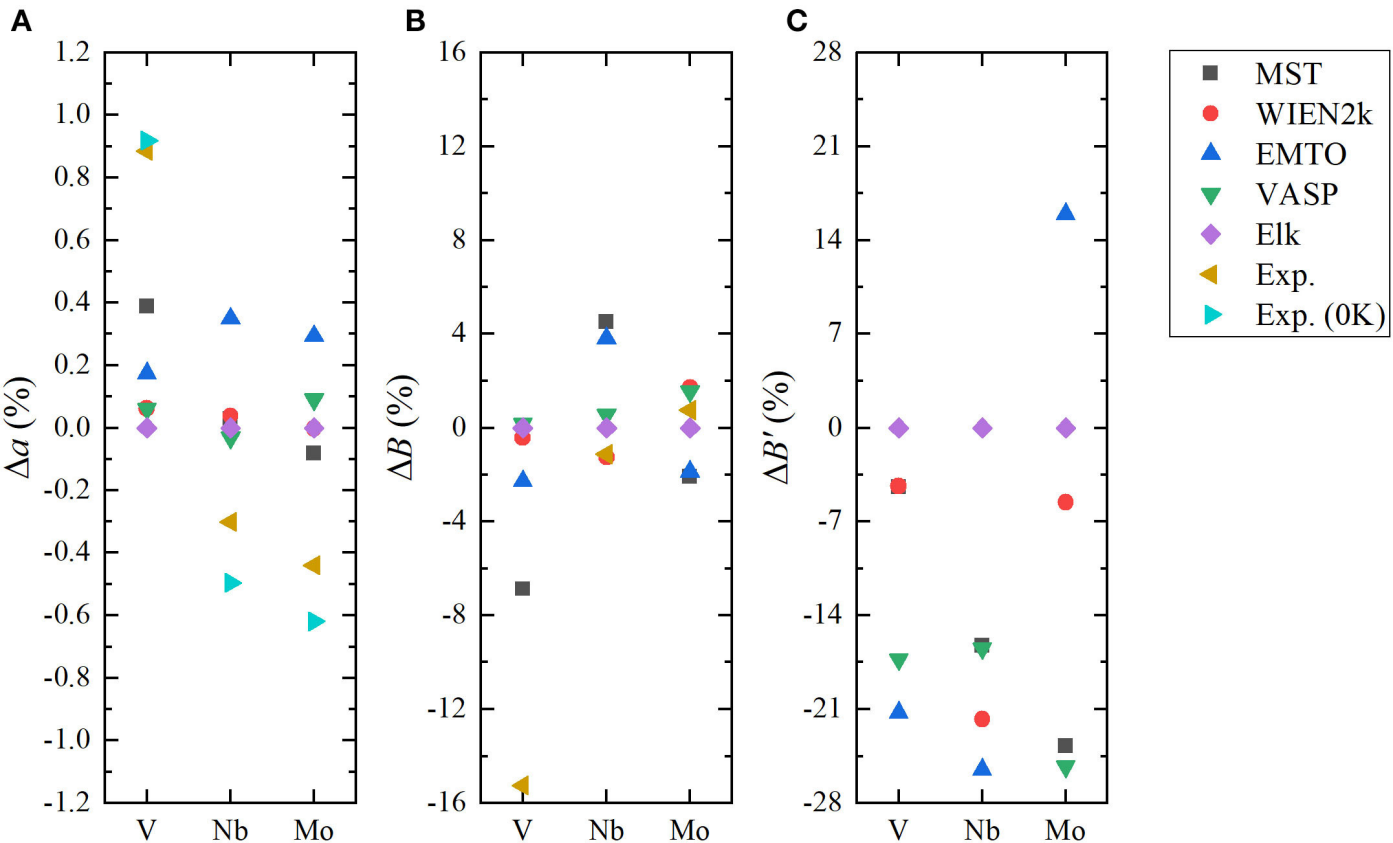
(Color online) Relative errors in lattice parameter **(A)**, bulk modulus **(B)**, and its derivation **(C)** (Δ*a*, *B*, Δ*B*′) for body-centered cubic (BCC) V, Nb, and Mo, where the Elk results are taken as reference values.

To investigate the accuracy of the FP MST method in the MuST package, we calculated *a*_0_, *B*_0_, and *B*′ of three bulk systems V, Nb and Mo, and compared the results with all-electron packages including WIEN2k, EMTO, and VASP. In these tests, we employed the same exchange-correlation functional and equivalent numerical settings, as introduced in section 3.1. Figure 1 shows the calculated *E*(*a*) curves from these packages, which have been shifted so that all *E*(*a*) points with the lowest energy are adjusted to zero. The fitted results of *a*_0_, *B*_0_, and *B*′ are given in Table 1. In addition, results from the Elk package, those obtained by the FP-LMTO method, and experimental values from the literature are also listed in Table 1 as a reference. The differences with respect to the results from Elk are illustrated in Figure 2.

**Table 1:** Equilibrium bulk properties [lattice parameter *a* (Bohr), bulk modulus *B* (GPa), and pressure derivative of the bulk modulus *B*′], the elastic constants $c^{\prime }=\left(c_{11}-c_{12}\right)/2,c_{11},c_{12}$, and *c*_44_(GPa) for body-centered cubic (BCC) V, Nb, and Mo metals.

**Method**	**a**	**B**	***B*′**	***c*′**	***c*_11_**	***c*_12_**	***c*_44_**
				**V**			
MuST	5.685	170.32	3.72	60.42	250.88	130.04	52.46
WIEN2k	5.667	182.10	3.72	59.69	268.91	149.53	19.90
EMTO	5.673	178.73	3.06	79.72	285.03	125.58	51.61
VASP	5.666	183.18	3.22	60.71	264.13	142.71	20.50
Elk (Lejaeghere et al., 2016)	5.663	182.89	3.89	–	–	–	–
FP-LMTO (Landa et al., 2006a)	5.673	182.70	–	67.30	272.43	137.83	37.40
Exp.(Frederikse, 1972; Maschke and Levy, 1983; Young, 1991; Haas et al., 2009)	5.713 (5.715)	155.0	–	54.85 (57)	228.7	119.0	43.20 (46)
				**Nb**			
MuST	6.258	178.62	3.22	51.94	247.88	144.00	35.52
WIEN2k	6.258	168.77	3.01	47.87	232.60	136.85	14.88
EMTO	6.278	177.44	2.86	74.07	276.20	128.06	51.00
VASP	6.254	171.87	3.21	50.02	238.56	138.52	16.62
Elk (Lejaeghere et al., 2016)	6.256	170.92	3.84	–	–	–	–
FP-LMTO (Landa et al., 2006a)	6.270	170.7	–	63.9	225.9	128.1	25.5
Exp.(Frederikse, 1972; Ashkenazi et al., 1978; Young, 1991; Haas et al., 2009)	6.237 (6.225)	169	–	52.89 (60)	246.5	134.5	28.73 (31)
				**Mo**			
MuST	5.968	253.65	3.22	169.52	479.67	140.64	131.59
WIEN2k	5.973	263.47	3.99	147.45	460.07	165.17	103.15
EMTO	5.991	254.21	4.89	169.80	480.61	141.01	131.02
VASP	5.978	263.08	3.15	148.57	461.18	164.03	102.56
Elk (Lejaeghere et al., 2016)	5.973	259.07	4.22	–	–	–	–
FP-LMTO (Söderlind et al., 2000)	5.970	255	–	139	440	162	139
Exp.(Dickinson and Armstrong, 1967; Frederikse, 1972; Young, 1991; Haas et al., 2009)	5.947 (5.936)	261	–	152.95	463.7 (473.0)	157.8 (156.2)	109.2 (110.9)

*Exp. stands for experimental results. The experimental values in parenthesis are the experimental lattice constants corrected for the zero-point anharmonic expansion (ZPAE) and experimental elastic constants corrected at 0 K. The MuST, WIEN2k, EMTO, VASP, Elk, and FP-LMTO are different ab initio methods*.

We see from Table 1 and Figure 2 that except for the bulk modulus of V, differences in the calculated *a* and *B* results are less than 0.5 % and 5 %, respectively, which could be considered as small discrepancies between different codes (Holzwarth et al., 1997; Kresse and Joubert, 1999; Lejaeghere et al., 2016). The lattice parameter *a* of BCC V metal obtained by MuST is slightly larger than that of other *ab initio* methods, but the difference of *a* is only 0.39%, with respect to the Elk's result. For BCC Nb and Mo, both MuST lattice parameters are very close to those results from Elk. The relative error is 0.03% for Nb and 0.08% for Nb, respectively. Generally speaking, the PBE predicted lattice parameter is overestimated, that is, theoretical lattice parameter is usually larger than experimental values, whereas for V, all present *ab initio* lattice parameters listed in Table 1 are smaller than the experimental value at 0 K. But for Nb and Mo, the *ab initio* lattice parameters are slightly larger, with respect to their experimental values. The bulk modulus *B* represents the stress v.s. the volume expansion or compression. And its derivative *B*′ can be used to describe the anharmonic effect in the vibrating lattice. Comparing the calculated bulk moduli and their derivatives, we find that for BCC V metal the MuST *B* is slightly smaller, within 6.9%, than the Elk bulk modulus, while EMTO, WIEN2k, and VASP results agree well with each other. This is consistent with the fact that the MuST lattice constant is slightly larger, within 0.4%, than the Elk result, whereas the relative discrepancy is within 0.2% among the results of other codes.

Finally, it is necessary to mention that the energy-lattice curve of a solid is sensitive to the treatment of semi-core states. For example, Nb has core (1*s*, 2*s*, 2*p*, 3*s*, 3*p*, 3*d*), semi-core (4*s*, 4*p*), and valence (4*d*, 5*s*) states. Due to the limitation in the current implementation of MuST, only the 4*d*5*s* electrons of Nb are considered as valence electrons, and the semi-core states are treated as core states. The same treatment is imposed for the semi-core states of V (3*s*, 3*p*) and those of Mo (4*s*, 4*p*). In MST as well as in other all-electron methods including LAPW and EMTO, both the core and the valence states participate in the self-consistent iteration. The difference is that the core states are calculated using the spherical part of the crystal potential in the atomic sphere Singh and Nordström (2006). The wave function for each core state is confined and normalized within the sphere radius. In the case that semi-core states are treated as core states, since their charges are no longer well confined inside the atomic sphere, the so-called confinement error appears and a proper setting of the bounding sphere radius becomes important Asato et al. (1999). Different from MuST, in the WIEN2k calculations, a recommended separation energy of -6.0 Ry automatically defines the core- and band-states. Accordingly, both the semi-core and valence states of V, Nb, and Mo metals are treated as band states and solved using the full potential of the crystal. In the PAW method of the VASP package, the frozen-core approximation is used, so the core electrons will not participate in the self-consistent calculations. And the PAW atomic datasets including semi-core states for V, Nb, and Mo are provided by the VASP POTCAR library to be utilized for accurate calculations. The main point is that: the differences in the treatment of the semi-core states may cause noteworthy discrepancies in the calculated results, and we suggest that the semi-core states are allowed to be treated as band states in the future version of MuST.

### 3.3. Elastic Constants

**Figure 3:**
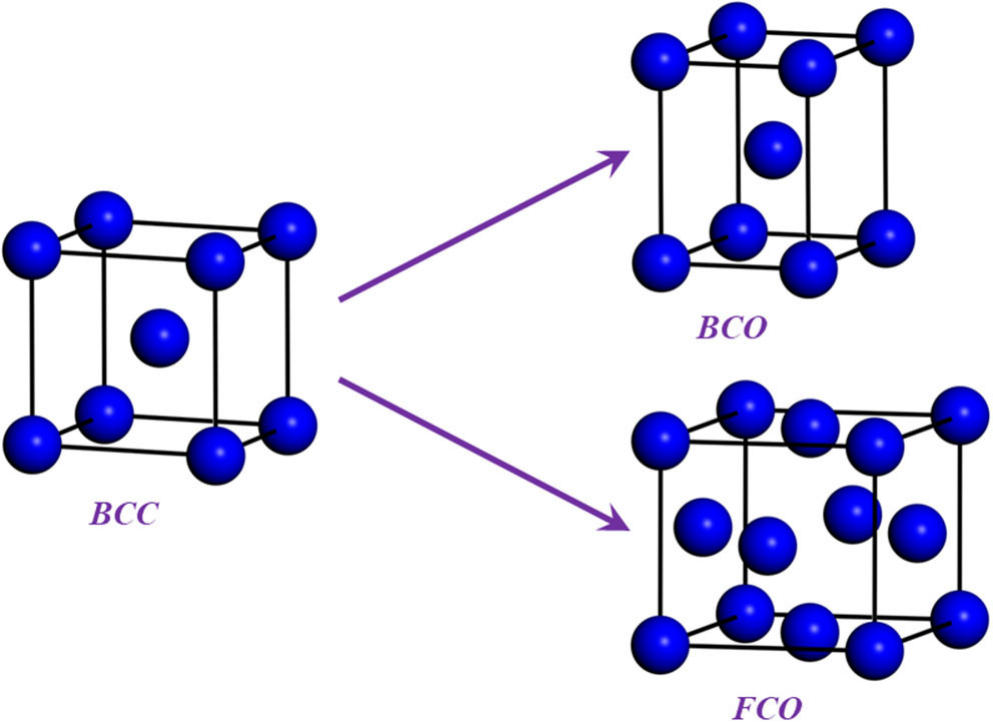
(Color online) The deformation configurations [body center orthorhombic (BCO) for the calculation of *c*′ and face center orthogonal (FCO) for the calculations of *c*_44_] for body center cubic (BCC) crystal.

In a cubic lattice there are three independent elastic constants, *c*_11_, *c*_12_, and *c*_44_, of which *c*_11_ and *c*_12_ are connected to the bulk modulus *B* = (*c*_11_ + 2*c*_12_)/3 and the tetragonal shear modulus $c^{\prime }=\left(c_{11}-c_{12}\right)/2$. The two shear elastic parameters *c*′ and *c*_44_ were computed according to the standard methodology (Vitos, 2007). For example, we used the following volume conserving orthorhombic and monoclinic deformations:

$$\begin{array}{l} \left(\begin{array}{ccc} 1+\delta_{o} & 0 & 0\\ 0 & 1-\delta_{o} & 0\\ 0 & 0 & \frac{1}{1-\delta_{o}^{2}} \end{array}\right)\text{ and }\left(\begin{array}{ccc} 1 & \delta_{m} & 0\\ \delta_{m} & 1 & 0\\ 0 & 0 & \frac{1}{1-\delta_{m}^{2}} \end{array}\right), \end{array}$$

which lead to the energy change $△E\left(\delta_{o}\right)=2V_{c}^{\prime }\delta_{o}^{2}+O\left(\delta_{o}^{4}\right)$ and $△E\left(\delta_{m}\right)=2V_{c44}^{\prime }\delta_{m}^{2}+O\left(\delta_{m}^{4}\right)$. Both energies were computed for six distortions, δ = 0.00, 0.01, …, 0.05. The body center orthorhombic (BCO) for *c*′ and faced center orthorhombic (FCO) for *c*_44_ are shown in Figure 3.

**Figure 4:**
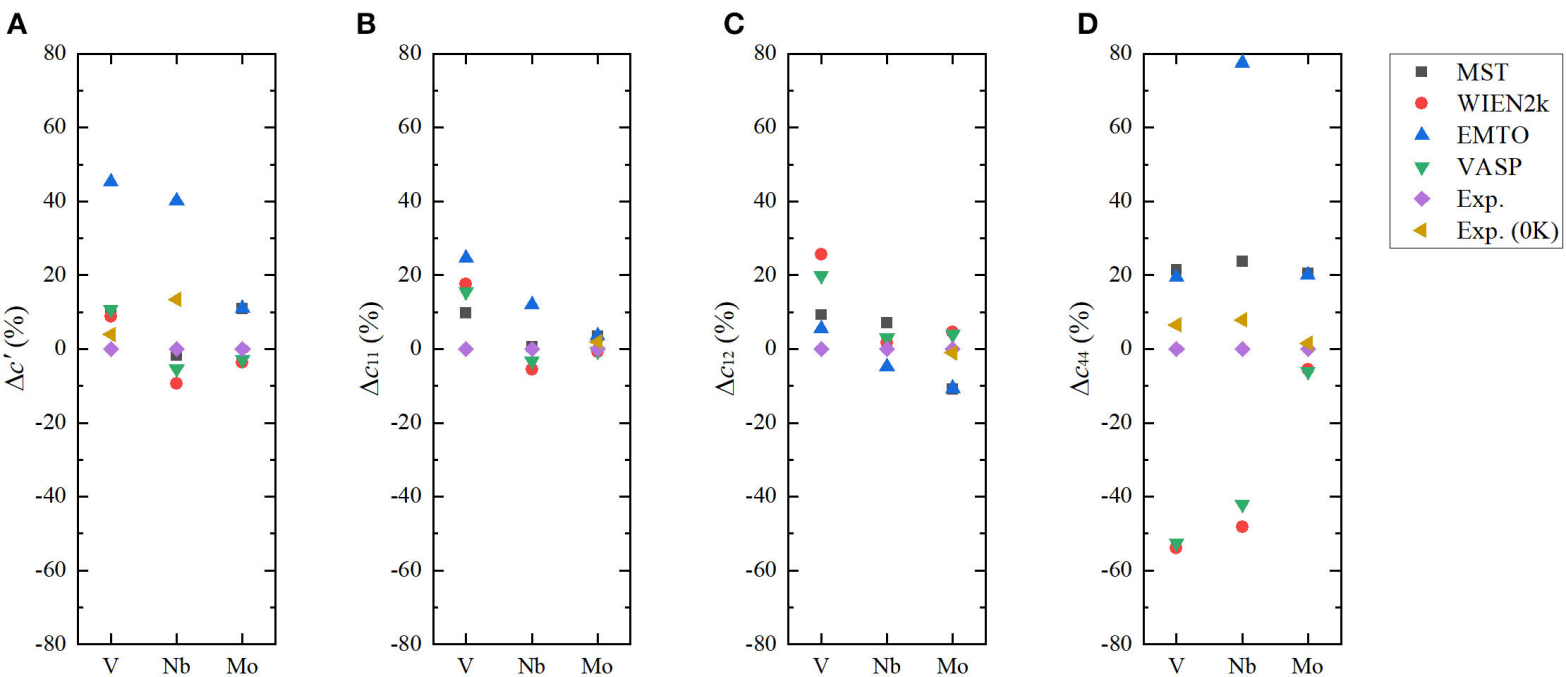
(Color online) Differences in the calculated *c*′ **(A)**, *c*_11_ **(B)**, *c*_12_ **(C)**, and *c*_44_ **(D)** from *ab initio* methods for body-centered cubic (BCC) V, Nb, and Mo with respect to experimental values at room temperature.

Results of elastic constants from different *ab initio* methods and experiments are listed in Table 1. Their differences with respect to experiments are shown in Figure 4. Due to the calculations of *c*_11_ and *c*_12_ via the combination of *c*′ and bulk modulus, the accuracy of *c*′ plays a key role in the quality of *c*_11_ and *c*_12_ results. From Figure 4, we can see that for the *c*′ of V, the MuST result agrees well with the results from WIEN2k and VASP. Due to the small bulk modulus, our MuST calculated *c*_11_ and *c*_12_ are slightly different from those of WIEN2k and VASP. For *c*_11_ and *c*_12_ of Nb, results from MuST, WIEN2k, and VASP are all close to experiments at room temperature, whereas the difference of *c*′ between calculations and experiments at 0 K is up to 13.4% for MuST, 20.2% for WIEN2k, and 16.6% for VASP. For Mo, the discrepancy of *c*′ with the experimental value is up to 11.0% for MuST and EMTO, but it is only 2.9%/3.6% for VASP/WIEN2k. This results in the large difference for *c*_11_ and *c*_12_ between MuST/EMTO and WIEN2k/VASP calculations. Although EMTO and FP-LMTO can be regarded as similar muffin-tin type methods, their calculated elastic constants are very different. The main reason is that the available FP-LMTO results were calculated based on the LDA (Söderlind et al., 2000).

From Table 1, we can find for V and Nb that *c*_44_ results of MuST and EMTO are close to experimental values, while those from WIEN2k and VASP much smaller. We note that the early work on elastic constants *c*_44_ is 17.1 GPa for V and 10.3 GPa for Nb (Koči et al., 2008; Nagasako et al., 2010). There is an anomalous dispersion of transverse acoustic phonons propagating along the <100> direction in V and Nb. Softening of acoustic phonons induces small values of the shear constant. The soft acoustic phonons and small shear constants are related to the nesting properties of the Fermi surface, which produce a van Hove singularity in the electronic DOS near the Fermi level (Landa et al., 2006b; Nagasako et al., 2010). Due to the presence of van Hove singularity, an extremely fine mesh for Brillouin zone integration suggested in Nagasako et al. (2010) was expected to determine the exact *c*_44_. However, in practice, the convergence of *c*_44_ with respect to the ***k***-point density may be very slow (Nagasako et al., 2010). Instead of using an extremely dense ***k***-mesh, the smearing methods can be used to handle the singularity in DOS. It is reported in Nagasako et al. (2010) that the smearing method has a clear impact on the *c*_44_ results. We note that smearing is performed in WIEN2k and VASP calculations, but in the MuST and EMTO codes no smearing methods are used. This might be the reason on the discrepancy between theoretical results. For Mo, the MuST calculated *c*_44_ is 18.7% larger than the experimental value at 0 K, but the *c*_44_ from WIEN2k and VASP are in good agreement with experiments. The ultimate reason of differences in the elastic constant between *ab initio* calculations and experiments is still far from resolved. We noted that there exist variations between experiment results, for example, for Mo the experimental value of *c*_44_ is about 110.9 GPa at 0 K reported in Dickinson and Armstrong (1967), while another experiment is about 125 GPa at 0 K (Featherston and Neighbours, 1963). So it may be necessary to estimate the accuracy of experiments at low temperatures and the improved extrapolation method may also be desirable.

## 4. Test on Scalability

In this section, we investigate the strong and weak scalabilities of the FP LSMS method implemented in MuST package.

### 4.1. Convergence on LIZ Size

**Figure 5:**
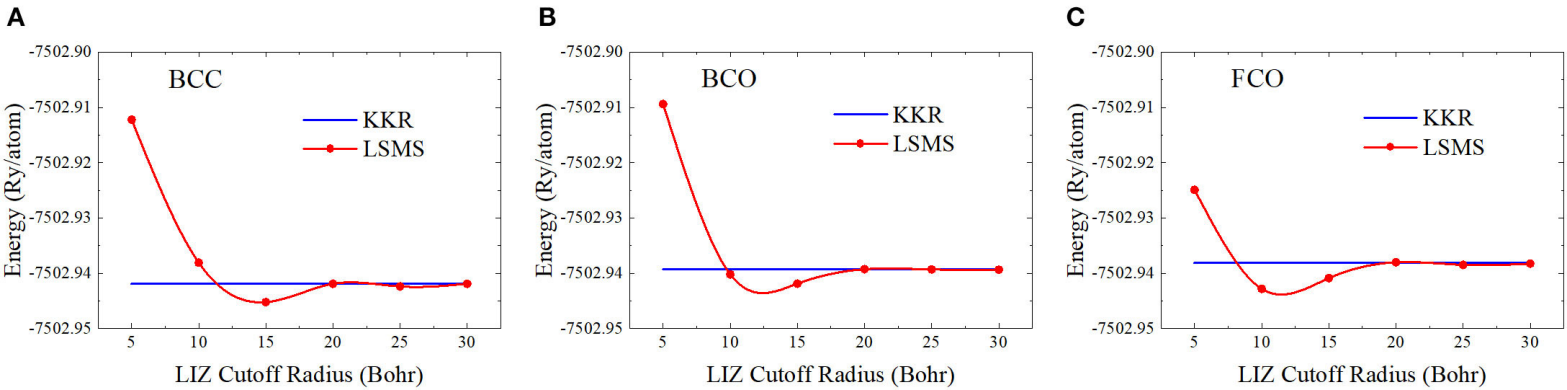
(Color online) Energy as a function of the local interaction zone (LIZ) radius for **(A)** body-centered cubic (BCC), **(B)** body center orthorhombic (BCO), and **(C)** faced center orthorhombic (FCO) structures, where the “KKR” stands for the results from the standard multiple scattering theory (MST) method.

In practice, the first question on the LSMS method may be the proper choice of the LIZ size for an atomic site. We can calculate the total energy of the bulk system using the LSMS method with increasing LIZ sizes and compare the results with those obtained by the standard MST method. The convergence tests on the LIZ radius have been performed for face-centered cubic (FCC) Cu and BCC Mo in Faulkner et al. (2018). For FCC Cu, the LSMS energy agrees with the reference MST result to better than 0.5 mRy when 6 neighboring shells are included in the LIZ. This corresponds to a cluster of 87 atomic sites with a LIZ radius of 11.7 Bohr. For BCC Mo, on the other hand, a larger LIZ is required in order to achieve better than 0.5 mRy accuracy. We test the convergence of the LIZ radius for BCC Nb and its deformed structures. As shown in Figure 5, the LSMS total energies converge to the MST results when the LIZ radius is larger than 20 Bohr. Indeed, we have achieved the accuracy of 0.5 mRy by including 14 neighboring shells into the LIZ, which corresponds to about 330 atoms. This is due to the fact that the Fermi energy falls in the *d* bands so that the DOS near the Fermi energy is significant.

We would like to mention that in the MuST code, the LIZ is embedded in the vacuum with free-electron GF. The LIZ size may be effectively decreased by choosing the effective medium instead (Zeller et al., 1995; Abrikosov et al., 1997), which could provide clues for improving the performance of the LSMS method in a future release.

### 4.2. Strong Scalability

The complexity of the FP MST method can be estimated by the weak scaling test. A good strong scalability is a prerequisite to an effective weak scaling test. Under the premise of good strong scalability, the computational overhead can be revealed by the execution time since the communication overhead contributes a small percentage. We constructed a BCC supercell consisting of 1024 niobium (Nb) atoms. The LIZ of each atomic site contains 89 atoms. As illustrated in Table 2, the LSMS method exhibits a good strong scalability. This is due to the two-level parallelism over atoms and energy points implemented in MuST package. The 1024 atoms are distributed over from 128 to 1024 MPI (message passing interface) processes. When the number of MPI processes exceeds the number of atoms, a second level of parallelization over energy points is performed.

**Table 2:** Strong scalability test of the full-potential multiple scattering theory (MST).

# MPI	128	256	512	1024	2048
Execution time (s) per SCF iteration	17581	8804	4855	2456	1255
Parallel efficiency (%)	100.0	99.8	90.5	89.5	87.6

**Figure 6:**
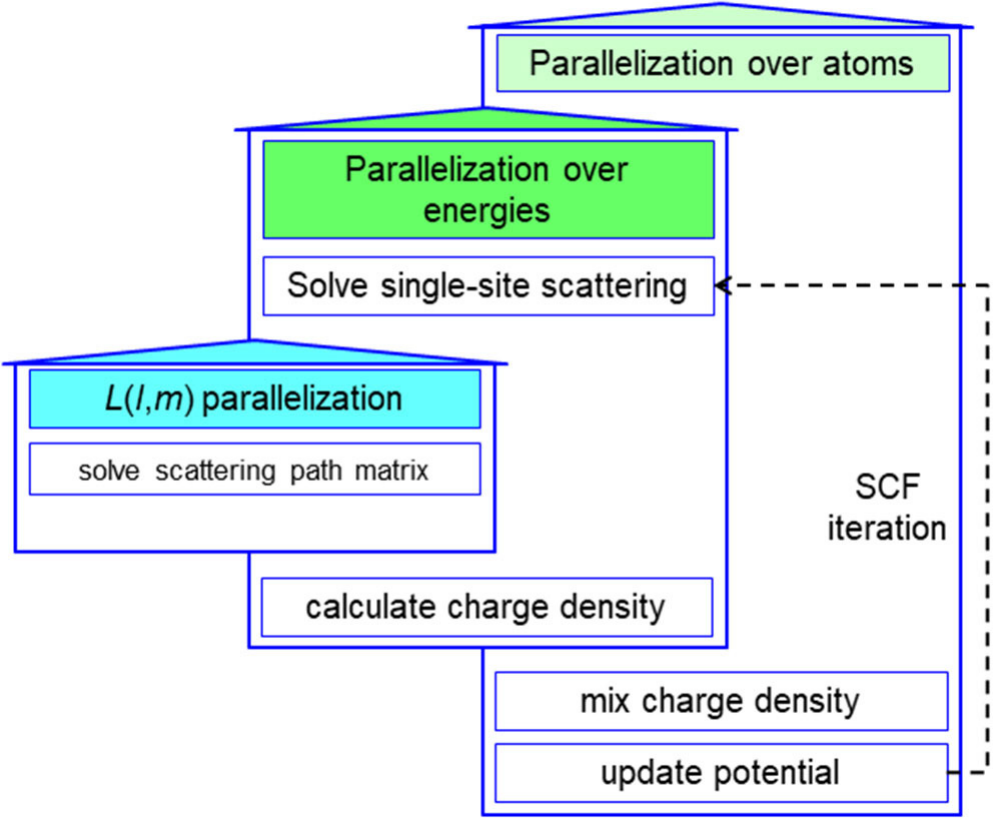
(Color online) A schematic illustration of highly parallelized multiple scattering theory (MST) method.

The intrinsic parallelism comes from the fact that the computation of the GF for each atom and each energy point along the complex contour is essentially independent. Each MPI process exchanges *t*-matrix with the others treating the neighboring atoms in the LIZ region. There are no global operations involved in the process of calculating the GF other than few global sum operations such as the summation of the net charge in each atomic cell for the determination of the electron chemical potential. Consequently, the MST method can be highly parallelized, as shown in Figure 6.

### 4.3. Weak Scalability

In the weak scaling test, the system size and the number of MPI processes are increased concurrently when one atom per MPI process is kept unchanged. In the pure atom parallelization, we observe a significant growth in the execution time with increasing atoms, as shown in Table 3. Consequently, the overall complexity of the FP MST method is not O(N). The execution time of one SCF iteration can be divided into two parts. One part is for the solution of the valence state by KKR-GF method. The other is used to update the effective potential. They are denoted by *t*_val_ and *t*_pot_ in Table 3. It can be observed that *t*_val_ remains almost the same while *t*_pot_ grows with increasing system size. So the linear scaling is achieved in solving the GF function, which is consistent with the tests in Thiess et al. (2012). As the system size becomes large, the computational overhead on updating the effective potential becomes gradually dominant, which deserves further analysis.

**Table 3:** Weak scalability test of the full-potential locally self-consistent multiple scattering (LSMS) method where *t*_scf_ stands for the execution time on one SCF iteration, *t*_val_ the execution time on calculating the valence states based on multiple scattering theory (MST), *t*_pot_ the execution time on updating the effective potential, *t*_xc_ the execution time on updating the exchange-correlation potential, and t^*^ to be defined in Section 4.4 is the execution time for an interpolation step in updating the Coulomb potential.

**# atoms**	***t*_scf_ (s)**	***t*_val_ (s)**	***t*_pot_ (s)**	t^*^ (s)	***t*_xc_ (s)**
64	391.45	249.28	141.86	120.84	19.94
128	517.29	252.65	263.03	241.74	19.79
256	818.45	253.18	562.11	539.58	20.46
512	1374.58	255.09	1115.19	1092.71	19.97
1024	2479.77	255.05	2216.79	2182.25	19.98

### 4.4. Scaling Analysis for Updating Potential

As shown in Table 3, the execution time on updating the exchange-correlation potential, denoted by *t*_xc_, remains almost the same as system size increases. Therefore, we concentrate on the Coulomb potential. In the FP method, the total charge density is divided into the following two parts:

(10) $$\begin{array}{l} \rho \left(r\right)=\tilde{\rho }\left(r\right)+\hat{\rho }\left(r\right), \end{array}$$

where $\tilde{\rho }$ is chosen as a smoothly varying density and $\hat{\rho }$ is the sphere-bounding non-overlapping charge density. The associated Coulomb potential with $\hat{\rho }$ can be formulated as like

(11) $$\begin{array}{l} {\hat{V}}_{\text{Coul}}\left(r\right)=2\int_{{\mathbb{R}}^{3}}\frac{\hat{\rho }\left(r^{\prime }\right)+\rho_{0}}{\left|r^{\prime }-r\right|}dr^{\prime }-2\underset{j}{\sum }\frac{Z_{j}}{\left|r-R_{j}\right|}, \end{array}$$

which can be calculated by the multi-pole expansion technique together with the periodic boundary condition and the constraint

(12) $$\begin{array}{l} \int_{{\mathbb{R}}^{3}}\hat{\rho }\left(r\right)dr+\rho_{0}\int_{{\mathbb{R}}^{3}}dr=\underset{j}{\sum }Z_{j}. \end{array}$$

The procedure is somewhat analogous to the calculation of the Coulomb potential in muffin-tin approximation. The difference is that the non-spherical potential in FP method has multi-pole expansion while the spherical one in muffin-tin approximation has only zero-order moment. Actually, both the two schemes have linear scaling.

The charge density $\tilde{\rho }$ can be regarded as a pseudo electron density varying smoothly. The associated Coulomb potential can be determined by solving the Poisson equation:

(13) $$\begin{array}{l} -\nabla^{2}Ṽ\left(r\right)=4\pi \tilde{\rho }\left(r\right). \end{array}$$

And fast Fourier transform (FFT) is used for solving Equation (13). In the MST method, both the electron density and one-electron potential are discretized on the spherical mesh around each atom. Therefore, an interpolation from the uniform FFT mesh to the spherical mesh is required. More specifically, the radial part Ṽ_*L*_ is calculated from $\tilde{\rho }$ on the uniform FFT grids. The computational scheme can be formulated as the integral form:

(14) $$\begin{array}{l} {Ṽ}_{L}\left(r_{j}\right)=\frac{2}{\pi }{\left(-ı\right)}^{l}\int_{{\mathbb{R}}^{3}}\tilde{\rho }\left(r^{\prime }\right)F_{L}\left(r_{j},r_{j}^{\prime }\right)dr^{\prime }, \end{array}$$

where $F_{L}\left(r_{j},r_{j}^{\prime }\right)$ is defined as follows:

(15) $$\begin{array}{l} F_{L}\left(r_{j},r_{j}^{\prime }\right)\equiv \int_{{\mathbb{R}}^{-3}\backslash \left\{0\right\}}\frac{1}{|G{|}^{2}}j_{l}\left(|G|r_{j}\right)Y_{L}^{*}\left(\hat{G}\right){\text{e}}^{ıG\cdot r_{j}^{\prime }}dG, \end{array}$$

where *j*_*l*_ is the spherical Bessel function and *Y*_*L*_ is the spherical harmonic. In Equations (14) and (15), *r*_*j*_ stands for the radial grid point, ***r***′ and ***G*** represent the real-space FFT grid and the corresponding reciprocal grid, respectively, and $r_{j}^{\prime }=|r^{\prime }-R_{j}|$.

**Figure 7:**
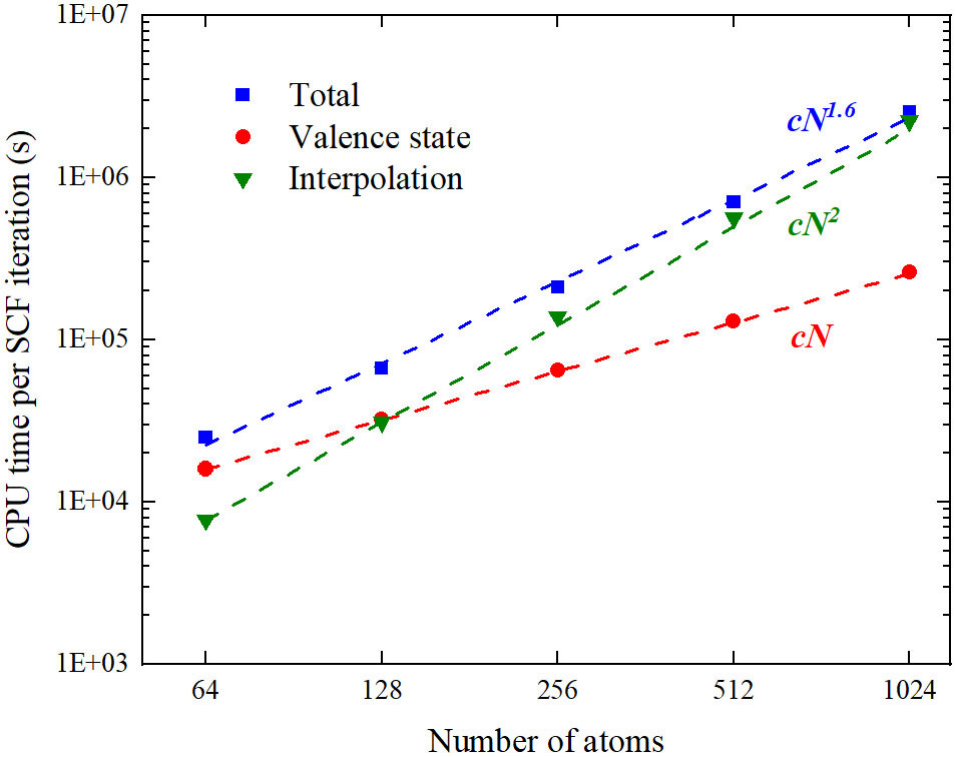
(Color online) The log–log diagram of CPU time vs. system size where the CPU time is the product of the execution time by the number of MPI processes.

Since $F_{L}\left(r_{j},r_{j}^{\prime }\right)$ is independent of the electron density, it can be setup once before the SCF iteration. However, the summation in Equation (14) scales as *N*_rad_ · *N* · *N*_FFT_, where *N*_rad_ is the number of radial grids and set to be 2001 in the test. And *N*_FFT_ is proportional to the number of atoms *N*, which yields $O\left(N^{2}\right)$ scaling in performing the interpolation like Equation (14). We locate the code segment to perform (14) and denote its execution time by t^*^ in Table 3. As shown in Figure 7, the calculations of valence states scale as O(N), while the interpolation from FFT uniform grid to the atom-centered radial grid exhibits an $O\left(N^{2}\right)$ scaling, which results in an overall scaling of $O\left(N^{1.6}\right)$. Therefore, a more efficient algorithm to update the Coulomb potential in angular momentum expansion is critical to achieve a linear scaling FP MST method.

## 5. Conclusion

We have investigated the accuracy and scalability of the FP MST method implemented in MuST package. The MST predicted lattice parameter for V, Nb, and Mo are consistent with the other calculations and the available experiments. The MST predicted bulk moduli, pressure derivative of the bulk modulus, and the *c*′ elastic constant are acceptable, expect for a relatively larger difference in the bulk modulus of V. While for *c*_44_, there exists large difference between theoretical and experimental results, the possible reasons have been discussed in details. It is suggested that a proper treatment of the semi-core states should be considered in the future version of the MuST package.

A significant advantage of the MST method is the reduced scaling in the calculations of metallic systems. Although the linear scaling has been reported previously under the muffin-tin approximation, tests in this work imply that the overall scaling of the FP method is not O(N). It is suggested that the updating of the Coulomb potential in angular momentum expansion should be further improved. Nevertheless, a favorable scaling as $O\left(N^{1.6}\right)$ can be achieved in the full-potential MST method, compared to the $O\left(N^{2}\right)$ to $O\left(N^{3}\right)$ scaling of frequently-used methods. Another advantages in MuST is the treatment of chemical and magnetic disorders based on the CPA.

In summary, the FP MST method shows the potential to simulate more complicated materials on massively parallel supercomputers. And MuST provides a reliable and accessible way to large-scale first-principle simulations of metals and alloys.

## Data Availability Statement

All datasets presented in this study are included in the article/supplementary material.

## Author Contributions

All authors listed have made a substantial, direct and intellectual contribution to the work, and approved it for publication.

## Conflict of Interest

The authors declare that the research was conducted in the absence of any commercial or financial relationships that could be construed as a potential conflict of interest.
